# Cognition is … Fundamentally Cultural

**DOI:** 10.3390/bs3010042

**Published:** 2013-01-04

**Authors:** Andrea Bender, Sieghard Beller

**Affiliations:** 1 Department of Psychology, University of Freiburg, Engelberger Straße 41, D-79085 Freiburg, Germany; 2 Department of Human Sciences, University of Paderborn, Warburger Str. 100, D-33098 Paderborn, Germany; E-Mail: sieghard.beller@uni-paderborn.de

**Keywords:** cognition, culture, computer metaphor, numerical cognition, ethnobiological reasoning, theory of mind

## Abstract

A prevailing concept of cognition in psychology is inspired by the computer metaphor. Its focus on mental states that are generated and altered by information input, processing, storage and transmission invites a disregard for the cultural dimension of cognition, based on three (implicit) assumptions: cognition is internal, processing can be distinguished from content, and processing is independent of cultural background. Arguing against each of these assumptions, we point out how culture may affect cognitive processes in various ways, drawing on instances from numerical cognition, ethnobiological reasoning, and theory of mind. Given the pervasive cultural modulation of cognition—on all of Marr’s levels of description—we conclude that cognition is indeed fundamentally cultural, and that consideration of its cultural dimension is essential for a comprehensive understanding.

## 1. Introduction

“As a cognitive scientist, I am only interested in the universality of cognition.” This position was advocated by more than one of the previous chairs of a society for cognitive science in Europe. While perhaps not held by cognitive scientists in general, this attitude is widespread, and its proponents may feel justified in their view due to how the field was conceptualized in previous decades. We argue that the focus on the universal aspects is not only short-sighted as a research strategy, and all too often not sufficiently substantiated, but that it is also significantly misleading. Cognition is fundamentally cultural, and excluding this dimension necessarily impedes its understanding and investigation.

This is hardly new [1], and yet it is widely ignored [2]. The vast majority of studies are based on rather small samples of participants from industrialized societies (predominantly from North America, Europe, and Australia), whose representativeness for “the human mind” is surely questionable [3]. These samples are “weird” in a double sense: *WEIRD* in the sense of their specific features, *W*estern, *E*ducated, *I*ndustrialized, *Ri*ch, and *D*emocratic; and *weird* in the sense of constituting a psychological outlier in the global comparison [4]. Generalizations from findings with these samples are therefore fragile to say the least. If the interesting aspects of cognition were indeed universal, this sampling habit would be non-problematic; any type of person should be as appropriate a subject as any other. The key question, of course, is: How do we know that the cognitive processes studied by cognitive scientists are indeed universal if we do not investigate this [5]?

As authors with backgrounds in cognitive psychology and cognitive anthropology, the perspective we put forward on what cognition might be and how its scientific investigation should be re-focused will be inevitably partial and subjective. As our point of departure, we take a notion of cognition that emerged during the 1950s and 1960s, and that is still popular in the field in which we work [6,7,8].

## 2. The Cognitive (Psychology) Perspective

Since the cognitive revolution laid the foundation for a new field [9,10,11], its (sub-)disciplines have been striving to achieve a comprehensive understanding of hu­man thinking and acting, and they do so from a perspective that focuses on cognition as information processing. Cognition in this sense encompasses many and diverse aspects such as perception, attention, categorization, learning and memory, thinking, decision making, problem solving, and language use [6,7,8,12]. All of these processes are linked to reason and are thus considered to be exemplars of *cold cognition*. Psychological process­es, which contribute to *hot cognition* such as emotion and motivation, have substantial cognitive aspects as well. The cognitive appraisal of a situation or event, for instance, is essential for eliciting specific emotions and for motivating a corresponding action [13]. Cognitive processes, in turn, may be accompanied and shaped by emotions—a phenomenon known as “motivated reasoning” [14,15]. 

The cognitive perspective, which we address, is based on a computational theory of the mind. This focuses on mental states of all kinds, which are generated and altered by way of information input, processing, storage, and transmission [16,17]. Such cognitive processes can be described on three levels [18,19]:

- The *computational* level refers to what and *why* something is done (e.g., addition as a means of combining items);
- The level of *representation and algorithm* describes *how* this is done (e.g., by using Arabic digit notation and beginning addition from the final position); and
- The level of physical *implementation* specifies *whereby* this is done, that is, the device that implements computational function, representation, and algorithm (e.g., a cash register or abacus).

Most of cognitive psychology has focused on the middle level and, more specifically, on the cognitive processes as the (presumably) most universal part of cognition. The remainder of this paper explores how these processes are shaped by culture.

## 3. Reconsidering the Nature of Cognition

The cognitive perspective, like the underlying computer metaphor, affords a range of assumptions, three of which will be highlighted here: (1) Cognition takes place internally, in people’s heads; (2) processing can be distinguished from content; and (3) processing is independent of context or people’s cultural background [20]. In the following, we explicate each of these assumptions, their implications and consequences for research on human cognition, and we illustrate these with one example each.

### 3.1. External Representations Affect (Internal) Processes

Cognitive psychology originated as a counter-movement to behaviorism, which categorically dismissed mental constructs as inaccessible to direct observation and thus as a subject for scientific inquiry. In contrast, the new discipline realized that behavior could only be accounted for if attention and information processing are considered. This focus on mental phenomena, however, was coupled with a disregard for external factors. A wide range of cognitive activities are performed in interaction with cultural artifacts, designed for the very purpose of facilitating cognitive processing [21], such as writing systems, numerical notation, maps, or navigational instruments [22,23,24,25]. With information being spread over internal and external representations, cognitive processing must be interactive and integrative, a feature captured by the term “distributed cognition”. One of the core findings in this field of research is that the properties of the external representation may affect how it is processed and that, therefore, different representations of the same abstract structure may cause different cognitive behaviors [22,23,25,26,27]. Such *representational effects* are particularly well investigated for numerical systems. 

Numerical systems consist of a more or less structured counting sequence and can be implemented in diverse modalities: as body-based systems [28], as verbal systems based on number words [29,30], and as notational systems based on written symbols or other external representations [25,31,32,33,34]. Independent of their implementation, each counting sequence constitutes a system with distinct structural properties. With regard to each of these properties, number systems may vary cross-culturally [35,36,37] and may entail implications for the processing of numbers. 

One critical property of notational systems in this regard is *dimensional representation*, illustrated in the following for two-dimensional base-10, that is, decimal systems. The base dimension (*i.e*., the basic numerals from 1 up to 9) can be represented by quantities or by symbols. The power dimension (*i.e*., the multiples of 10, 100, and so on) can be represented in a *parsed* manner (with two distinct symbols for multiplier and power term, as in the number word “two hundred”), in an *integrated* manner (with a single symbol for both multiplier and power term, as in the Greek letter σ), or in a positional manner (in which the power level is indicated by position only, as in 200). Dimensional representation determines whether base and power dimension are externally separable (as in parsed systems) or only internally separable (as in integrated systems). It also determines the extent to which numerical information is explicated: Whereas a symbolic representation explicates category (*i.e*., nominal information only), a quantitative representation also explicates magnitude (*i.e*., ordinal, interval, and ratio information). This has straightforward effects, for instance, on the cognitive load in numerical tasks: Explicit in­formation is perceptually available, whereas implicit information needs to be retrieved from memory, thereby increasing the cognitive load [25,38]. Whether two-digit numbers are compared in a holistic, parallel, or sequential manner also crucially depends on how these numbers are represented [26].

Depending on such properties, numerical systems do have distinct effects, for instance on working memory, number processing, and task difficulty, as has been demonstrated in conceptual analyses [24,35,36,37,38], simulation models [25,33], and empirical studies [39,40,41]. This highlights the relevance of external aspects for internal cognitive processes, and not only in terms of how smoothly such processes may operate, but also which processes are activated in the first place. External representations, by their very nature, are culture-dependent and culture-specific, even if they are as widespread as the Arabic digit notation. Findings on effects of number representation on number processing therefore also demonstrate how susceptible cognitive processes may be to cultural influences.

### 3.2. Content Affects Processing

As noted above, the primary approach in cognitive psychology to describe and understand human thinking and behavior focuses on information processing: It sees people as mentally representing information and as processing these representations in one way or another [8,16]. This corresponds to Marr’s middle level of description, the level of representation and algorithm [18,19]. However, the bulk of empirical research is concerned more with the processual part than the representational part, largely based on the assumption that these processes could—or even should—be scrutinized irrespective of content. Content in the form of background knowledge is conceived of as varying indi­vidually and as blurring the findings strived for in psychological tasks [42,43,44,45,46]. However, cognitive processes are typically tailored to a specific format of representation; the format of the data (*i.e*., its representation) determines which algorithms (*i.e*., processes) can be performed easily and elegantly, and which cannot [47]. Teasing apart content and process is therefore no simple task [48], and ignoring content in investigations of cognitive processing has produced misleading results and incorrect conclusions, as will be demonstrated for inductive reasoning. 

Inductive reasoning is concerned with drawing general conclusions from specific observations [49,50]. One of the key questions addressed in the respective research is concerned with the strength of inductive arguments [51]: How readily do people generalize from a single observation? In order to examine this question, people are presented with two arguments of the following kind:

(a) “Robins have sesamoid bones; therefore birds have sesamoid bones.”
(b) “Penguins have sesamoid bones; therefore birds have sesamoid bones.”

and are then asked which of these arguments they consider to be a more reliable generalization. Most people choose (a) as more certain [51]. Robins are considered to be prototypical birds [52], and typical category members are taken more readily as a source for generalizations. On the basis of such results, process models of inductive reasoning such as the *similarity coverage model* [51] and the *feature-based model* [53] have been developed, which use similarity between categories as the core explanatory variable.

Typically, studies of this kind use categories that are familiar to participants (e.g., robins, penguins, and birds); the property to be generalized, however, employs so-called ‘empty’ predicates, that is, predicates for which people possess no content-specific knowledge such as “sesamoid bones”. Empty predicates help researchers to analyze the causal effects of the relationship between categories, irrespective of the content of the property to be generalized, but inevitably lead to an underestimation of the role that content-specific knowledge may play. This is exacerbated by the sample of students with an urban background, who often possess little and only theoretical knowledge about animals and plants.

Allowing for complex background knowledge changes the picture. Studies on inductive reasoning with students from the US and bird experts both from the US and Guatemala (indigenous Itzaj Maya) were able to replicate the typicality effect (as well as the related diversity effect), but only for the students; in the two expert groups, the allegedly robust effects evaporated [54,55]. In contrast to the students, these experts possess broad causal and ecological knowledge, on which they drew extensively for their inductive judgments. These findings demonstrate that the specific type of content under consideration—here: categories and their similarity (in the case of the students) *vs*. categories in the context of a broader causal model (in the case of the experts)—determines which cognitive processes are triggered and which inductive generalizations are drawn. Process models that consider only similarity (such as the two models mentioned above) therefore inevitably fall short [56].

A thorough examination of cognitive processes must take into account content. Research on expertise, both within the biological domain [57,58,59,60] and beyond [61,62], has con­vincingly shown the great extent to which such expertise modifies the organization of knowledge and thereby the manner in which a given task is solved—an effect so profound that it contributes even to a reorganization of brain structures [62,63,64,65,66].

Content effects are not constrained to expertise, however, as can be illustrated, again, with an example from biological reasoning. A prevailing assumption in developmental psychology is that intuitive biological reasoning emerges in an anthropocentric manner from intuitive psychological reasoning [67,68]. This assumption is based on a series of tasks on inductive reasoning in which children up to the age of seven years regard biological phenomena in analogy to psychological phenomena. Developing an understanding similar to that of adults requires a conceptual change and occurs at a later age. However, cross-cultural comparisons between Menominee children from a Native American reservation and children from both a rural and an urban Euro-American population shook this assumption: Only the urban children initially hold an anthropocentric perspective—that is, children who possess rich experiences with humans, but not with the natural environment. The observed conceptual change can thus not be regarded as a general developmental pattern. In addition, the two rural groups of children also differed, and in this case, responsible for this finding is not a difference in their experiential background, but in their cultural background [60,69]. The role of these more general background influences will be explicated in the next section.

### 3.3. Cultural Habits and Practices Affect Processing

While an impact of content and its representational format can be accounted for by Marr’s model—and may thus be acceptable even to those scholars emphasizing universality—a more general impact of culture from outside cognitive domains appears less acceptable. Yet, cultural habits and practices of a more general nature can also be shown to have an impact on how information is processed.

People do not simply reason, they *learn* to reason. The way in which one pays or shifts attention, the aspects upon which one focuses, the extent and depth of relations and interaction one takes into account all depend, at least to some extent, on how other people in one’s group attend, focus, and take into account. The spectrum of processes affected by such influences is broad, ranging from basic levels of visual perception, through economic decision-making, ethnobiology and spatial cognition, to self-concept and various social psychological phenomena [4,5], and similar variability can be observed for the assumed linguistic universals [70]. Cognitive domains for which differences across cultures and languages have been documented in previous years include, among others, the susceptibility to optical illusions such as the Müller-Lyer illusion or the Sander Parallelogram illusion [71,72], attentional processes [73,74,75], the way in which people represent space [76,77,78], time [79,80,81,82,83], or number [37,40,84,85,86], or how they attribute and infer causal effectiveness [87,88,89]. To illustrate this, let us turn to a fundamental cognitive capacity, the theory of mind.

Theory of mind refers to the insight that other people have knowledge, beliefs, and desires that may differ substantially from one’s own knowledge, beliefs, and desires. This insight emerges in children between age four and five—and apparently largely irrespective of potentially influencing factors [90]. The theory of mind constitutes an essential prerequisite for most social interactions, but it also serves as foundation for our folk-psychological models, that is, our assumptions on what motivates people, why they respond to an event in the way they do, or how social harmony could be ensured [91]. The substantial differences in folk psychologies across cultures raises the question of how these presumptions may interfere with the development of the theory of mind in children. While it is beyond doubt that all children do develop a theory of mind, the age at which this takes place is more variable than has previously been assumed [92,93,94,95]. Whether this difference is one of performance only or also of competence, and whether it is due to interaction patterns between parents and children, due to linguistic features, or simply due to problems with the experimental setting, remains a topic of debate [91,92,95,96,97,98,99]. However, the empirical evidence accumulated so far suggests that language plays a crucial role in raising awareness of mental states, and in particular the availability and frequency of usage of *Mentalese* vocabulary (*i.e*., terms for mental states such as thinking, wanting, or understanding). The Junín Quechua, for instance, are very reluctant to speculate on others’ mental states, and terms for cognitive processes are hardly ever used. This may explain why Junín children take longer to infer what others may think or know [92]. The same is true for some groups in Oceania, where others’ mental states are considered to be opaque and to some extent untouchable [95,100,101].

## 4. Conclusions

The cultural diversity of cognition is one of the most controversial topics in cognitive psychology (and perhaps cognitive science more broadly). For a long time, any potential of culture to affect cognition was as vigorously denied as was a potential of language to affect thought (the underlying principle of linguistic relativity, also known as Sapir-Whorf hypothesis [102,103,104,105,106,107], is still hotly debated [76,77,78,108,109,110,111,112], but its renewed investigation has opened the door for a rich field of research [113,114]). Yet, as we have tried to demonstrate above, culture does affect cognition, and it does so in various ways and on all three of Marr’s levels of description. On the level of representation and algorithm (upon which we focused here), internal cognitive processes are affected by external, culture-specific representations; they are affected by the culture-dependent content to be processed; and they are affected more generally by people’s cultural background. Cultural influences on cognition are presumably even stronger on the computational level, where the “what” and “why” of cognition is reflected [18]. There, the context of both reasoning and research into reasoning are broadly affected by how people interpret a task and how they choose to respond to it [19,115,116]. Even on the implementation level, culturally shaped experiences and expertise feed back onto the very structure of the brain [62,63,64,65,66,117], thus “re-engineering the mind” [62]. These findings justify the conclusion that culture is fundamental to cognition, not only as its origin [118] and enhancer [62], but also as an integral part of it [119,120,121,122,123,124,125]. 

This is not to say that cognition has no universal aspects. In fact, more aspects than not may well be universal—whether due to the cognitive architecture or the way in which the world in which we live is structured. However, only a small number of the assumed psychological universals can be regarded as sufficiently established [4,5]. Teasing apart the universal and the culture-dependent aspects must therefore remain a fundamental goal for cognitive psychology and for the cognitive sciences more generally, if they are still striving to achieve a comprehensive understanding of human cognition.
